# Study on Characteristics of Electromagnetic Coil Used in MEMS Safety and Arming Device

**DOI:** 10.3390/mi11080749

**Published:** 2020-07-31

**Authors:** Yi Sun, Wenzhong Lou, Hengzhen Feng, Yuecen Zhao

**Affiliations:** 1 National Key Laboratory of Electro-Mechanics Engineering and Control, School of Mechatronical Engineering, Beijing Institute of technology, Beijing 100081, China; louwz@bit.edu.cn (W.L.); fenghengzhen@gmail.com (H.F.); zhaoyc0911@163.com (Y.Z.); 2 Beijing Institute of Technology, Chongqing Innovation Center, Chongqing 401120, China

**Keywords:** micro-electro-mechanical systems safety and arming device, electromagnetic coil, electromagnetic force, Maxwell energy balance formula

## Abstract

Traditional silicon-based micro-electro-mechanical system (MEMS) safety and arming devices, such as electro-thermal and electrostatically driven MEMS safety and arming devices, experience problems of high insecurity and require high voltage drive. For the current electromagnetic drive mode, the electromagnetic drive device is too large to be integrated. In order to address this problem, we present a new micro electromagnetically driven MEMS safety and arming device, in which the electromagnetic coil is small in size, with a large electromagnetic force. We firstly designed and calculated the geometric structure of the electromagnetic coil, and analyzed the model using COMSOL multiphysics field simulation software. The resulting error between the theoretical calculation and the simulation of the mechanical and electrical properties of the electromagnetic coil was less than 2% under the same size. We then carried out a parametric simulation of the electromagnetic coil, and combined it with the actual processing capacity to obtain the optimized structure of the electromagnetic coil. Finally, the electromagnetic coil was processed by deep silicon etching and the MEMS casting process. The actual electromagnetic force of the electromagnetic coil was measured on a micro-mechanical test system, compared with the simulation, and the comparison results were analyzed.

## 1. Introduction

As one of the key components of ammunition, the miniaturization and intelligence of the fuse is of great significance for weapon systems. A smaller-sized fuse can provide additional space for micro-sensors, micro-actuators, and other electronic devices [1,2,3,4]. The traditional safety and arming device can fulfill the functions of safety and arming required by the fuse; however, the large size and numerous components of safety and arming devices create issues—such as difficulty in assembly, low precision, and poor anti-overload—which seriously affect the performance of the fuse [5,6]. The micro-electro-mechanical systems (MEMS) safety and arming device has been studied due to its small size, light-weight, and high anti-overload capabilities. The U.S. Army Armament Research, Development and Engineering Center has conducted research on MEMS fuse technology and produced smaller, safer, and less expensive safety and arming devices than ever before [7,8,9,10,11].

Three main methods are used to fabricate MEMS Safety and Arming devices. The first way is the ‘lithographie, galvanoformung, abformung’ (LIGA) method, which is based on metal substrates [12,13,14,15]. In this method, a MEMS metal spring and slide are needed, and the structures are set perpendicular to each other to form an interlocking mechanism. 

Such devices can function well when driven by proper set back and rotation acceleration, and as most of the metals are elastic materials, they also have good explosion-proof characteristics [16,17,18]. However, the metal structures fabricated by LIGA are quite expensive. Although some researchers have used electroplating to replace LIGA, the large structure size and complex process method still limits the development of device miniaturization [19,20]. The second fabrication technique is the pyrotechnics method [21,22,23,24]. Based on micro-pyrotechnics, Robinson proposed a novel MEMS safety and arming device early in 2005, setting two MEMS springs perpendicular to each other to form an interlocking mechanism. This device can also function well when driven by proper set back and rotation acceleration [25,26,27]. However, using MEMS springs fabricated by LIGA are difficult to assemble. The V-shaped beam MEMS safety and arming device developed by Xi’an Jiaotong University, which functions under an electric heating environment with a driving voltage at 11 V [28], can achieve reliable function in electric heating, but this device requires larger energy module to operate—such as a Tantalum capacitor with voltage capacity of 16 V and a volume of 2.88 cm^3^—which has relatively large size and is not conducive to the miniaturization and integrated package of the system. Another disadvantage of this device is its high working voltage, which generates too much heat in the working process, wherein the excessive temperature can detonate the explosive by mistake. An electromagnetic driver of a safety and arming device designed by Nanjing University of Science and Technology [29] has a large displacement (3 mm) and sufficient electromagnetic force (3–50 mN). However, the size of the electromagnetic driver is 13×13×20 mm, which is too large for miniaturization and integration of systems. Another electromagnetically driven MEMS safety and arming device was also developed by Nanjing University of Science and Technology by using the fabrication method of UV-LIGA process based on SU-8 adhesive [30]. The maximum driving force of the lock pin of the two sets of safety and arming devices produced was 10 mN and 18 mN, and the maximum driving force of the MEMS slider was 13 mN, which could withstand impact acceleration of more than 20,000 g. However, the large size of the electromagnetic coil takes up too much space in this device.

In this paper, a new micro electromagnetically driven MEMS fuse safety and arming (safety and arming) device is presented. As a key component of the MEMS safety and arming device, the impact release mechanism is designed parametrically and fabricated using silicon bulk micromachining technology. Two electromagnetic coils are embedded in the MEMS safety and arming device. The coils are manufactured by deep silicon etching and a die-casting process. The security mechanism conforms to the idea of integrated design, which not only meets the requirements of an intelligent and miniaturized MEMS fuse, but also reduces the space occupied by the fuse security system and improves the reliability of the system. The ultimate device size is successfully minimized to 15 × 9 × 0.5 mm. The electromagnetic coil is the most important driving component in the MEMS safety and arming device. Due to the small size and precise structure of the electromagnetic coil, when increasing the electromagnetic force of the electromagnetic coil as much as possible in the design, we also need to consider the factors of large current, high temperature, and processing technology of the electromagnetic coil. Therefore, we first carried out theoretical calculation on the electromagnetic coil model. Parametric simulation of the model was then conducted to optimize the structure of the model. Finally, it was verified by the experiment.

## 2. Design and Simulation

### 2.1. Working Principle

**Figure 1:**
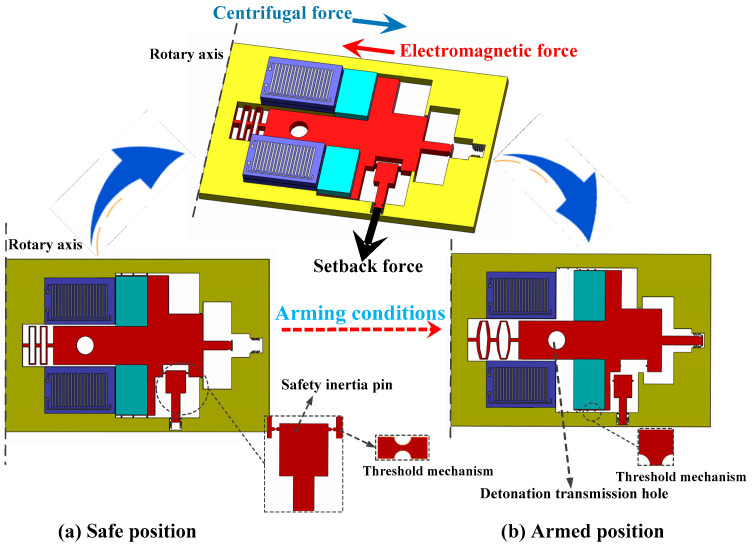
Working principle of MEMS safety and arming device.

A schematic of the silicon-based MEMS Safety and Arming device (15 × 9 × 0.5 mm) is shown in Figure 1. The MEMS safety and arming device is used mainly for rotary ammunition. As shown in Figure 1, the silicon-based MEMS safety and arming device is placed parallel to the rotary axis. Under the condition of a launch overload, the safety inertia pin, threshold mechanism, and the slider achieve arming status sequentially. First, the threshold mechanism at the connection between the safety inertia pin and the main slider breaks under the setback overload, and the safety inertia pin moves to the bottom, where it is locked by the locking mechanism. At the same time, electromagnetic force generated by the switched on coil overcomes centrifugal force to limit the movement of the main slider. When the release condition is satisfied, the electromagnetic coil is powered off, and the threshold mechanism between the main slider and frame breaks under the centrifugal force. The slider moves in the direction of the centrifugal force and is finally limited to a predetermined position by a locking mechanism, whereupon the detonation transmission hole is aligned with the electric detonator. At this point, the MEMS fuse is armed.

### 2.2. Design of Electromagnetic Actuator

**Figure 2:**
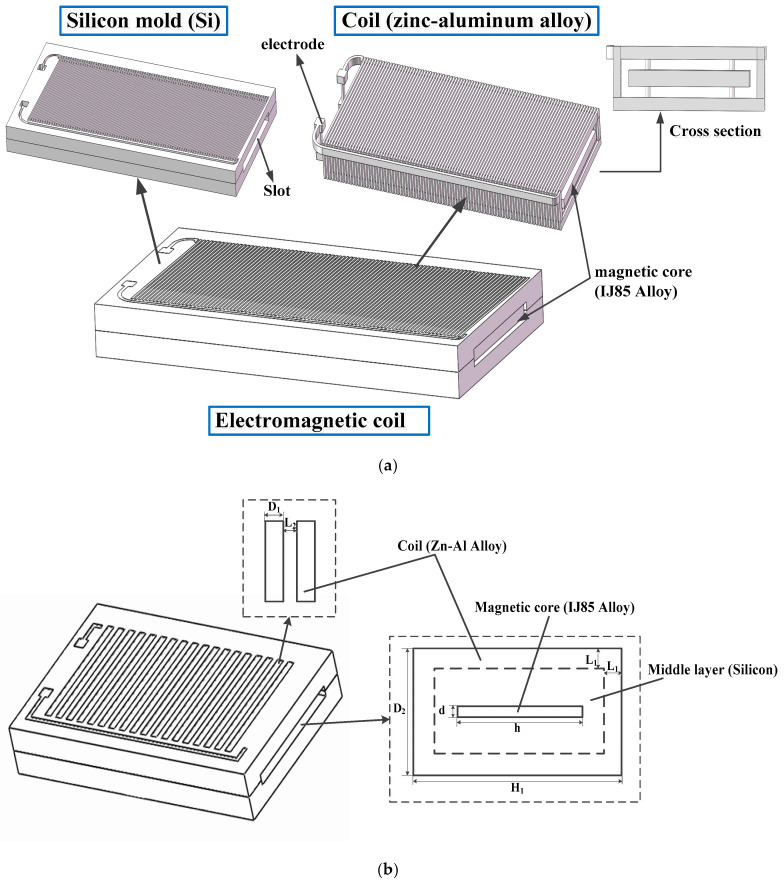
Integral structure diagram and cross section image of the electromagnetic coil. (**a**) Silicon mold, coil and composite model of electromagnetic coil. (**b**) A schematic of the electromagnetic coil.

**Figure 3:**
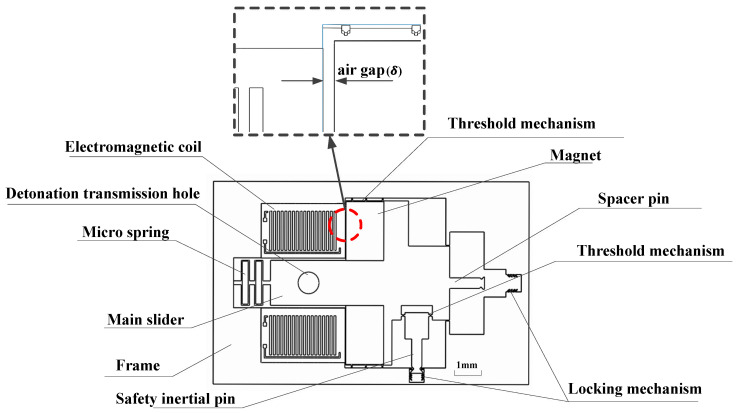
Schematic of MEMS safety and arming device.

The overall view and parameters of the designed electromagnetic coil are shown in the Figure 2, Figure 3 and Table 1. The working principle of the electromagnetic coil is that the magnetic core made of soft magnetic material is aligned with and magnetized by the magnetic field generated by the switched on coil. This strengthens the generated magnetic field and exerts a magnetic force onto the magnet which is bonded to the slider of the safety and arming device with a bonding adhesive. It can be seen in the Figure 2a, the electromagnetic coil was formed by casting zinc-aluminum alloy into a silicon mold, which was made of deep silicon etching. Additionally, the silicon mold is divided into two substrates, which are bonded together by bonding adhesive. From the cross-section of the coil, it can be seen that there is a layer of silicon between the wire of the coil and the magnetic core. The wire was made out of a zinc-aluminum alloy whose material properties are close to copper but with higher resistivity and better resistance to current, so that it produces less heat in the process of working than copper. The magnetic core inserted in the slot, as shown in Figure 2a, was a long strip type, and the material was IJ85 quaternary alloy with high permeability. The material parameters of the electromagnetic coil are shown in Table 2 and the current voltage of the electromagnetic coil was 5 V.

### 2.3. Theoretical Calculation

The Maxwell energy balance formula is used to calculate the electromagnetic force because of the large working air gap (δ/d>0.2 or δ/h>0.2, δ see Figure 3). According to the principle of virtual displacement we can obtain

(1) $$F_{x}=-\frac{1}{2}F_{mm}^{2}\frac{d\Lambda_{\delta }}{d\delta }$$

where the negative sign indicates that the electromagnetic force F is pointing in the direction of the decreasing air gap, in other words, the electromagnetic force is suction. At the same time, air gap permeability also needs to be modified

(2) $$G_{\delta }=\frac{\mu_{0}}{\delta }\left(d+K\delta \right)\left(h+K\delta \right)$$

where $F_{mm}$ represents the magnetomotive force and $F_{mm}=NI$, ***N*** represents the number of coil turns and ***I*** represents the current intensity, $\Lambda_{\delta }$ represents the air gap permeability, and K=0.31/π.

When the air gap is evenly distributed and leakage permeability does not change with the air gap, we can obtain

(3) $$\frac{d\Lambda }{d\delta }=-\mu_{0}\frac{S}{\delta^{2}}$$

where *S* is the cross-sectional area of the magnetic flux.

Since the air gap between the magnet and the electromagnetic coil is large, the magnetic resistance of the magnetic core and non-working air gap can be ignored, under the condition of constant magnetomotive force. Thus, Formula (1) can be written as

(4) $$F_{x}=\frac{1}{2}{\left(IN\right)}^{2}\frac{d\Lambda_{\delta }}{d\delta }$$

If the pole edge effect of air gap flux is excluded, Formula (4) can be changed to

(5) $$F_{x}=\frac{1}{2}{\left(IN\right)}^{2}\mu_{0}\frac{S}{\delta^{2}}=\frac{1}{2}{\left(IN\right)}^{2}\mu_{0}\frac{d\times h}{\delta^{2}}$$

It can be seen that the electromagnetic force is inversely proportional to the square of the air gap value under the condition of constant magnetomotive force.

Electromagnetic coil resistance can be calculated as

(6) $$R_{xq}=\rho \frac{l_{xq}}{S_{xq}}$$

(7) $$l_{xq}=2\times \left(D_{1}+H_{1}\right)\times N_{1}$$

(8) $$S_{xq}=D_{1}\times L_{1}$$

Then, the magnetomotive force can be obtained based on Formulas (6)–(8)

(9) $$NI=N1\frac{U}{R_{xq}}=N_{1}\times \frac{U\times S_{xq}}{\rho \times l_{xq}}$$

Electromagnetic force can be determined by substituting Formula (9) into (5).

## 3. Simulation and Discussion

**Figure 4:**
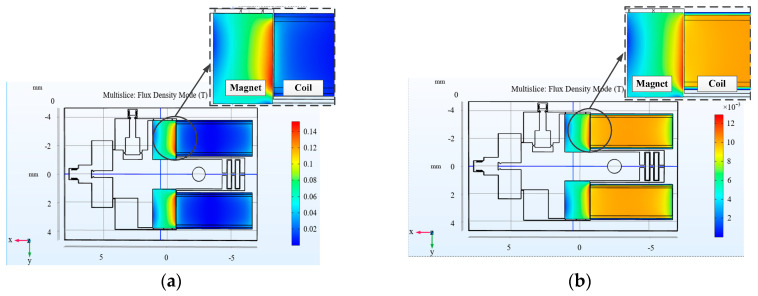
Finite element simulation of the electromagnetically driven safety and arming device. (**a**) Finite element simulation of electromagnetic coil with magnetic core. (**b**) Finite element simulation of electromagnetic coil without magnetic core.

The finite element simulation of the electromagnetically driven safety and arming device was carried out using COMSOL multiphysics field simulation software and the simulation image is shown in Figure 4. The simulation mainly calculated the electromagnetic force, resistance, current, and electric power of the electromagnetic coil at 5 V. In the simulation structure, the wire width, wire thickness, wire clearance, thickness of the magnetic core, and coil turns of the electromagnetic coil were 40, 100, 25, 100, and 90 μm. In Figure 4, the coil with the magnetic core inserted is shown on the left, which means there is a long strip type magnetic core in the electromagnetic coil, while on the right there is no magnetic core, only air. It can be seen from the figure that the edge of the magnet close to the coil has the highest magnetic flux density. Meanwhile, by comparing the two figures, it can be found that the maximum magnetic flux density between the coil, which has magnetic core, and the magnet is more than 10 times that of the coil without a magnetic core. In addition, our simulation calculation shows that the electromagnetic force between the coil with magnetic core is 0.0174 N, while the force between the coil with no magnetic core and the magnet is $6.62\times 10^{-5}$ N.

The simulation data of resistance, electric current, electric power, and electromagnetic force were compared with the theoretical calculation. The comparison data are shown in Table 3. It can be seen from the table that there was almost no difference between the theoretical calculation and simulation.

**Figure 5:**
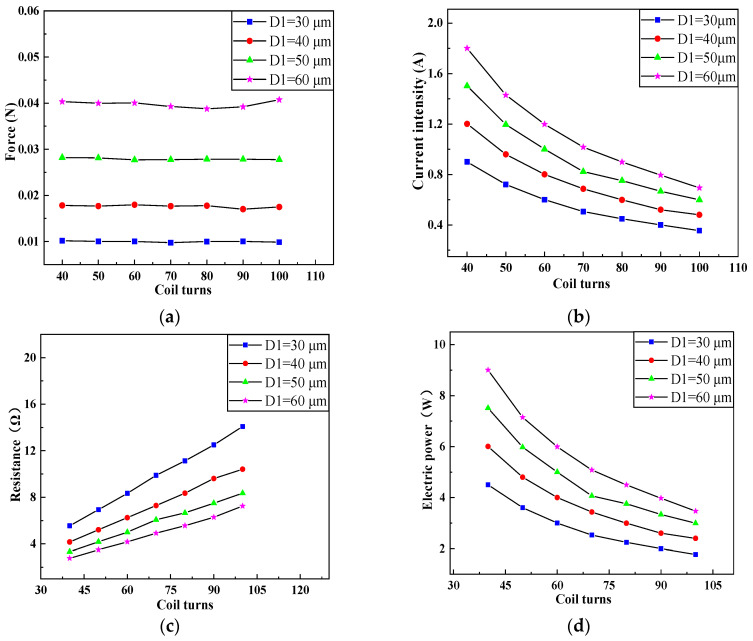
Variation of parameters of the electromagnetic coil with coil turns under different wire width. (**a**) Electromagnetic force of the electromagnetic coil with coil turns under different wire width. (**b**) Current intenstity of the electromagnetic coil with coil turns under different wire width. (**c**) Resistance of the electromagnetic coil with coil turns under different wire width. (**d**) Electric power of the electromagnetic coil with coil turns under different wire width.

Therefore, we used COMSOL multiphysics to build a parametric model of the electromagnetic coil, and analyzed the influence of coil turns on the electromagnetic force, current, resistance, and electrical power of the electromagnetic coil when the wire width of the coil was 30, 40, 50, and 60 μm respectively. Parametric simulation was carried out on the model, the results are shown in Figure 5.

As illustrated in the figure, under the same wire width of the electromagnetic coil, electromagnetic force and resistance of the coil increased with an increase in the number of coil turns, while the coil current and electric power displayed opposite trends. The electromagnetic force, coil current, and coil power increased with the growth of coil width, while the coil resistance decreased. We can also see that the increase of electromagnetic force with the number of coil turns was not obvious. However, due to the limitations of the overall size of the MEMS safety and arming device and the current resistance of the electromagnetic coil, it is expected to control the current below 1 A and the electric power below 4 W, while simultaneously increasing the electromagnetic force as much as possible. It was observed that the coil worked best when the wire width was 40 μm and the number of coil turns was 90.

**Figure 6:**
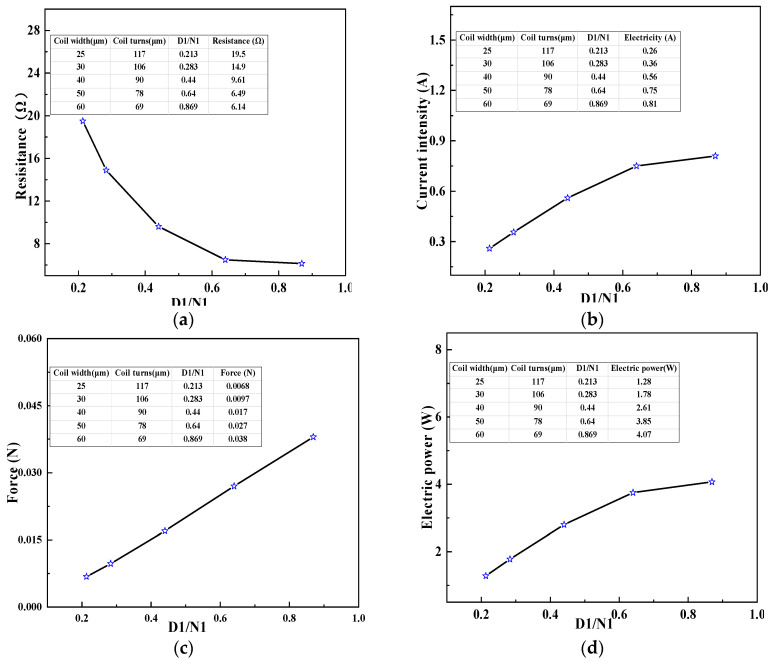
Influence of wire width and coil turns on the parameters of the electromagnetic coil. (**a**) Influence of wire width and coil turns on Resistance of the electromagnetic coil. (**b**) Influence of wire width and coil turns on Current intensity of the electromagnetic coil. (**c**) Influence of wire width and coil turns on Electromagnetic force of the electromagnetic coil. (**d**) Influence of wire width and coil turns on Electric power of the electromagnetic coil.

In this paper, the overall length of the designed electromagnetic coil was no more than 6 mm. If the wire width of the electromagnetic coil increased, the number of coil turns decreased, given that the total length of the coil was fixed. However, the effect of the wire width and coil turns on the electromagnetic force was the opposite. Therefore, it was necessary to analyze the influence of the coupling of wire width and number of coil turns on the electromagnetic force, resistance, current, and electric power of the electromagnetic coil. We set the wire clearance *L*_2_, wire width *D*_1_, and the number of coil turns *N*_1_ as 25, 40, and 90 μm, respectively. At this point, the total coil length *L*_3_ = (*D*_1_ + *L*_2_) × *N*_1_ = 5.85 mm, then the corresponding number of coil turns was calculated when the wire width of the coil was 30, 50, and 60 μm. Figure 6 shows the relationship between the ratio of D1 and N1 of electromagnetic force, current, and electric power.

As illustrated in Figure 6, as the value *D*_1_/*N*_1_ increased, that is, with the growth of the wire width D1, the number of coil turns decreased, and the electromagnetic force, current, and power increased obviously. This indicates that when the wire width and the number of coil turns change at the same time, the influence of wire width on the coil parameters plays a major role. It can be seen from Figure 6 that when the wire width of the coil was 40 μm and the number of coil turns was 90, it not only met the size requirement of the electromagnetic coil for the MEMS safety and arming device, but also meets the requirement that the current was less than 1 A and the electric power was less than 4 W.

Table 4 shows the influence of wire clearance on the parameters of the electromagnetic coil under different wire width and the wire clearance increased from 20 μm to 35 μm. We can obtain from Table 4 that the effect of wire clearance of the coil on the current and electric power was very small, and can be almost ignored.

This is because the resistance of the coil is related to the resistivity, length, and the cross-sectional area of the wire, but not to the wire clearance.

**Figure 7:**
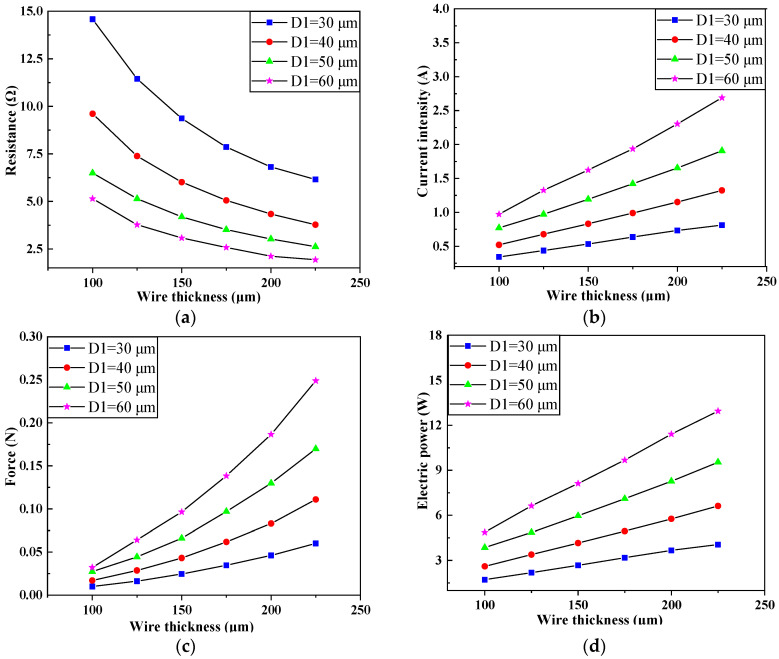
Influence of wire thickness on the parameters of the electromagnetic coil under different wire width. (**a**) Influence of wire thickness on the Resistance of the electromagnetic coil under different wire width. (**b**) Influence of wire thickness on the Current intensity of the electromagnetic coil under different wire width. (**c**) Influence of wire thickness on the Electromagnetic force of the electromagnetic coil under different wire width. (**d**) Influence of wire thickness on the Electric power of the electromagnetic coil under different wire width.

**Figure 8:**
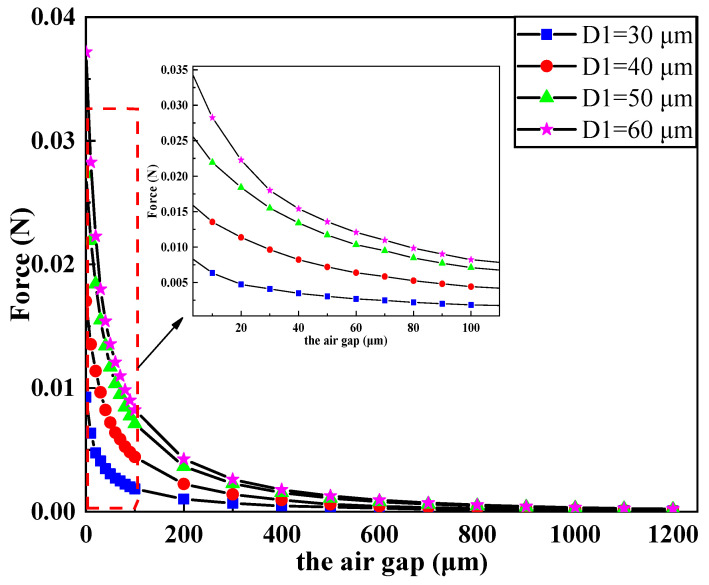
Influence of the air gap on parameters of the electromagnetic coil under different wire width.

Figure 7 shows the influence of wire thickness on coil resistance, current, electromagnetic force, and electrical power when *D*_1_ was 30, 40, 50, and 60 μm, respectively. As can be seen from Figure 8, when the wire width and the number of coil turns remained unchanged, the resistance of the coil tended to decrease with the increase of the wire thickness, while the current, electromagnetic force, and electric power increased. At the same time, it can be seen that the larger the wire width, the lower the rate of change of resistance with the wire thickness, while the larger the rate of change of electromagnetic force, coil current, and electric power. This is because the resistance of the coil is $R_{xq}=\rho \frac{l_{xq}}{S_{xq}}$, and the cross-sectional area of the wire is $S_{xq}=D_{1}\times L_{1}$. The larger the wire width *D*_1_, the smaller the rate of change of resistance with wire thickness *L*_1_, and the change of resistance is directly related to the coil current, electromagnetic force, and electric power.

Figure 8 shows the influence of the air gap between the electromagnetic coil and the magnet on the electromagnetic force. It is shown in the figure that the electromagnetic force of the coil decreased with the increase of the air gap. In addition, we can also observe that the electromagnetic force changed significantly when the air gap was within 100 μm. With the increase of the air gap, the rate of change of electromagnetic force became increasingly smaller until it reached zero.

To summarize, based on the theoretical calculation of the electromagnetic coil and the parametric simulation of relevant geometric dimensions, combined with the actual process capacity, the structure design and optimization of the electromagnetic coil was completed.

## 4. Fabrication Process for the Electromagnetic Actuator

Since the slot depth to width ratio of die-cast metal in the electromagnetic coil was 17:1, which is a structure with high aspect ratio, this paper adopted deep reactive ion etching technology and bonding technology to prepare the electromagnetic coil. A-A’ and B-B’ represent the main and side views. 

**Figure 9:**
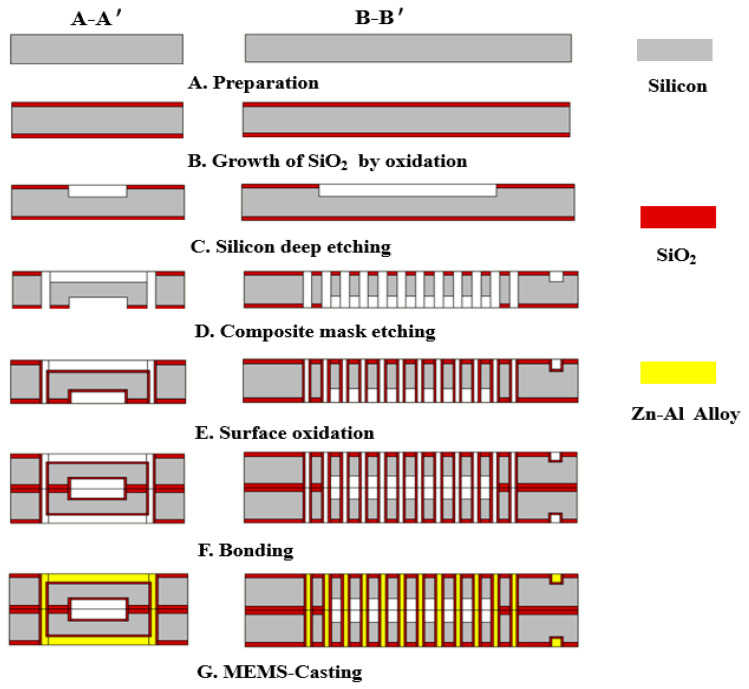
Process flow for the fabrication of the electromagnetic coil.

The eight steps of the manufacturing process of the electromagnetic coil structure used in this paper are shown in Figure 9.

**Figure 10:**
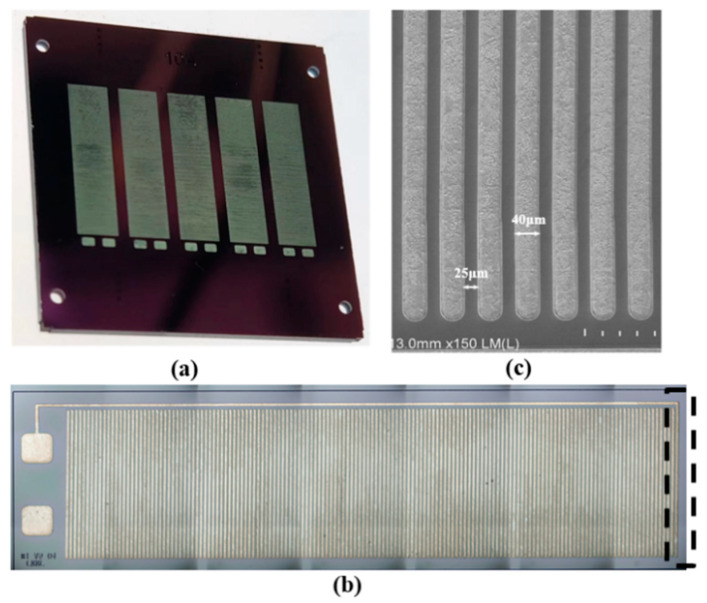
Structure of the electromagnetic coil (**a**) and scanning electron microscope images (**b**,**c**).

A. The Si (425 μm) substrate was prepared;

B. Silicon oxide of 500 nm thickness was grown on the Si substrate;

C. The magnetic core cavity was etched with deep silicon etching technology at an etching depth of 50 μm;

D. On the opposite side of the silicon wafer, the preparation of slots and holes was completed by composite mask technology;

E. The surface oxidation process was used to make an insulation layer with a thickness of 500 nm;

F. The structural bonds in step E were formed into a complete spiral coil mold using bonding adhesive;

G. The filling of the metal zinc-aluminum alloy of the spiral coil was realized using MEMS-casting™ technology to complete the preparation of the metal coils;

Figure 10a is the wafer diagram of the electromagnetic coil before cutting, and Figure 10 b,c are images of the electromagnetic coil under the electron microscope. It can be seen from the electron microscope images that the wire width of the coil was 40 μm, and the wire clearance between the single-turn of the coil was 25 μm. In addition, due to the cutting process, it can be seen from the dotted line in Figure 10b that there was a distance between the metal wire and the whole end of the coil, which may cause a gap between the electromagnetic force measured in the experiment and the theoretical calculation and simulation.

## 5. Electromagnetic Force Test

**Figure 11:**
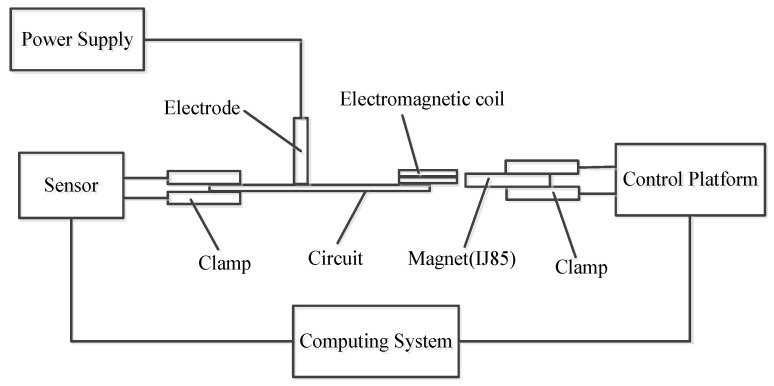
Schematic diagram of the electromagnetic coil testing system.

To test the mechanical properties of the electromagnetic coil and understand the difference between the experimental results of the electromagnetic coil and the theoretical simulation, we used the micro-mechanical test system to analyze the relationship between the magnitude of the electromagnetic force and the displacement. The test platform consisted of a power supply, force sensor, clamp, control platform, and computer system. The schematic diagram of the test system is provided in Figure 11.

**Figure 12:**
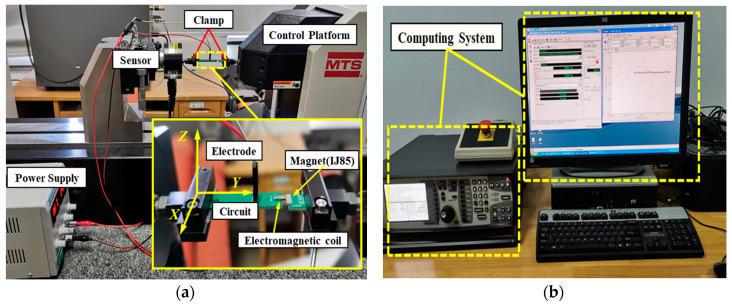
Photograph showing the components of the micro force testing system. (**a**) The control platform of the micro force testing system. (**b**) The computing system of the micor force testing system.

Figure 12 shows the experimental test image. As shown in Figure 12a, the electromagnetic coil was fixed on the circuit board by silver paste welding, the electrode was connected to the power supply through a wire, the circuit board was fixed by a clamp at one end, and the magnet IJ85 was fixed on the clamp at the other end. The control platform could be adjusted by the computer so that the cross-section of the magnet was aligned with the cross-section of the electromagnetic coil, and the distance between the magnet and the electromagnetic coil could be adjusted to the micron level. At the same time, the force sensor was connected to the computer system, and the image of electromagnetic force and displacement could be obtained according to the distance between the electromagnetic coil and the magnet.

The testing process of the electromagnetic coil was as follows:

(1) The system was powered on, and the supply voltage of the electromagnetic coil was 5 V;
(2) The computer system drove the control platform to align the cross section of the magnet with the center of the cross section of the electromagnetic coil. The initial distance was zero;
(3) The control platform was set by the computer system to drive the magnet to move in the Y direction, and the displacement was 2 mm, when the electromagnetic force was almost zero;
(4) The computer provided the image of the relationship between the electromagnetic force and displacement with an accuracy of 1 mN.

**Figure 13:**
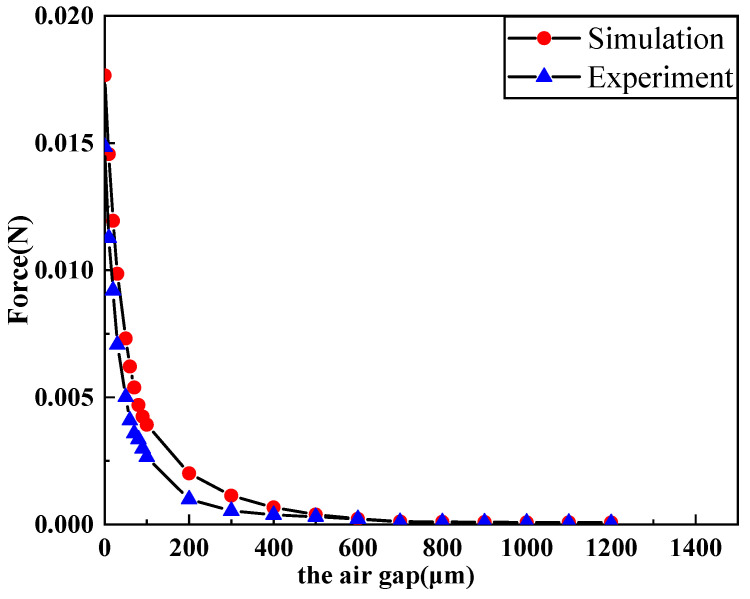
Comparison of experiment and simulation of electromagnetic force.

The extracted experimental data are plotted and compared with the simulation, the graph line was as follows:

As can be seen from Figure 13, the electromagnetic force measured in the experiment was smaller than the theoretical simulation value. One reason for this is that there was a small distance between the metal wire in the coil and the tail of the coil as a whole. It was also caused by magnetic flux leakage in the air gap during the experiment. However, as can be seen from Table 5, the difference between the current and the resistance was not significant, and was under the 5 V driving voltage.

## 6. Conclusions

In this paper, we presented an electromagnetically driven MEMS safety and arming device that was small in size, low in power consumption, and in line with the idea of integrated design. As an important driving component of the MEMS safety and arming device, the electromagnetic coil was small in size and precise in structure. To this end, we firstly designed and calculated the geometric structure of the electromagnetic coil and analyzed the model using COMSOL multiphysics field simulation software. The resulting error between the theoretical calculation and the simulation of the mechanical and electrical properties of the electromagnetic coil was less than 2% under the same size. A parametric simulation of the electromagnetic coil was then carried out and combined with the actual processing capacity to obtain the optimized structure of the electromagnetic coil. Finally, the electromagnetic coil was processed by deep silicon etching and the MEMS casting process. The actual electromagnetic force of the electromagnetic coil was measured using a micro-mechanical test system and compared with the simulation. The results showed slight differences, mainly due to the coil processing and air gap. Compared with the electro-thermal driven safety and arming device designed by Xi’an Jiaotong University, it needs a minimum 11 V drive voltage, but the output displacement is only 28.4 μm, which is too small for safety and arming devices. Meanwhile, the 11 V drive voltage needs to be matched with a large energy module, which is not conducive to the miniaturization and integration of the whole system. The electromagnetically driven MEMS safety and arming device of Nanjing University of Science and Technology has a driving voltage of 5 V and a large enough electromagnetic force (30–50 mN), but the size of the electromagnetic coil is too large (6 × 8 × 6 mm) to achieve miniaturization and integration. The electromagnetic drive S&A device designed in this paper has enough driving electromagnetic force (17–40 mN), small size of driving coil (2.5 × 6 × 0.8 mm), low drive voltage (5 V), small volume of power supply module, and can realize system miniaturization. How to improve the mechanical and electrical properties of MEMS electromagnetic coils and test the overall structure of the electromagnetic driven MEMS safety and arming device will be further studied in the future.

## Figures and Tables

**Table 1:** MEMS drive performance comparison.

Driving Type	Driving Principle	Displacement	Force	Security
Electrostatic drive	Electrostatic force	short	small	good
Electrothermal drive	Thermal stress	short	large	poor
Electromagnetic drive	Electromagnetic force	long	large	good

**Table 2:** Material parameters of the electromagnetic coil.

	Material	Silicon	Zn-Al Alloy	IJ85 Alloy
Property				
Magnetic permeability		1	1	250,000
Electrical conductivity (S/m)		1 × 10^−12^	1.5 × 10^7^	14.6
Relative permittivity		11.7	1	1
Saturation magnetic induction (T)		—	—	0.75

**Table 3:** Experiment and simulation data comparison.

	Theoretical Calculation	Simulation
Resistance (Ω)	9.75	9.6
Current intensity (A)	0.51	0.52
Electric power (W)	2.56	2.60
Electromagnetic force (N)	0.0178	0.0174

**Table 4:** Influence of wire clearance on the parameters of the electromagnetic coil under different wire width.

		Resistance (Ω)	Current Intensity (A)	Electric Power (W)	Force (mN)
30 μm	Min	12.72	0.39	1.95	9.8
	Max	12.88	0.41	2.05	10.2
	Average	12.81	0.4	2	10
	Std. deviation	0.08	0.01	0.05	0.2
40 μm	Min	7.78	0.66	3.3	27.2
	Max	7.92	0.68	3.4	28.7
	Average	7.85	0.67	3.35	27.9
	Std. deviation	0.07	0.01	0.05	0.08
50 μm	Min	7.78	0.66	3.3	27.2
	Max	7.92	0.68	3.4	28.7
	Average	7.85	0.67	3.35	27.9
	Std. deviation	0.07	0.01	0.05	0.08
60 μm	Min	6.14	0.81	4.05	37.7
	Max	6.47	0.77	3.85	39.4
	Average	6.3	0.79	3.95	38.6
	Std. deviation	0.13	0.02	0.1	0.11

**Table 5:** Experimental simulation data comparison.

	Resistance (Ω)	Current Intensity (A)	Electric Power (W)
Simulation	9.7	0.49	2.5
Experiment	9.83	0.50	2.46
